# 2023 Acknowledgment of *mBio* Invited Editors

**DOI:** 10.1128/mbio.03027-23

**Published:** 2023-12-19

**Authors:** Arturo Casadevall

**Affiliations:** 1 Johns Hopkins Bloomberg School of Public Health, Baltimore, Maryland, USA

## EDITORIAL

On behalf of the editors of *mBio*, I gratefully acknowledge the following individuals, who served as invited editors for the journal in 2023. While not members of the Board of Editors, invited editors serve an important role in the review process. Invited editors are experts in their fields of research who add an additional level of quality to the review process. An editor may assign a paper to an invited editor when they would like to have an additional expert opinion of the reviews or when the subject area falls outside the editor’s primary area of expertise.

Abram Aertsen

Jorge Alvarez

Katherine R. Amato

Asaad Mohammed Ataa

Eric Baehrecke

Hanna-Mari Baldauf

Alan D. Barrett

Christopher F. Basler

Himanshu Batra

Anthony D. Baughn

Patrik M. Bavoil

Victoria K. Baxter

J. David Beckham

Chase Beisel

Lena Biehl

Grant R. Bowman

Stanley Brul

Daniel H. Buckley

Scott Canfield

Rachel Carmody

James E. Cassat

Patricia A. Champion

Ewa Chrostek

Beatrice Clouet-d'Orval

Patrick Collins

Gino Corsini

Kyle C. Costa

Georgina Cox

Robert A. Cramer

Sean Crosson

Bryan R. Cullen

Anthony L. Cunningham

Ajai A. Dandekar

Katarzyna Danis-Wlodarc‌z‌yk

Daniel Dar

Matt Daugherty

Kristen M. DeAngelis

Maurizio Del Poeta

Nicole J. De Nisco

Gregory Q. Del Prete

Aravinda M. de Silva

Corrella S. Detweiler

Joseph P. Dillard

George Dimopulos

Shou-Wei Ding

Dirk P. Dittmer

Ray Dixon

Tobias Doerr

Maria Joanna Doligalska

Xiuzhu Dong

Jaquelin P. Dudley

Jonathan Dushoff

Melissa Ellermann

Joel Ernst

Oliver T. Fackler

Ying Fang

Paul D. Fey

Adriana Forero

Annette Fox

Michael Freitag

Matthew B. Frieman

Xinmiao Fu

Takema Fukatsu

Benjamin Gewurz

Daniel E. Goldberg

Ido Golding

Alan G. Goodman

Jeffrey A. Gralnick

Karen Guillemin

James C. Gumbart

Suryaram Gummuluru

John S. Gunn

Haitao Guo

Maximiliano Gutierrez

Ted Hackstadt

Mike Hadfield

Neal D. Hammer

Reuben S. Harris

Steven D. Harris

Sophie Helaine

Emily Hemann

Birgitta Henriques-Normark

Russell T. Hill

Stacy M. Horner

Wei-Shau Hu

Yilin Hu

Jianming Hu

Johannes Huebner

Bruce A. Hungate

William T. Jackson

Steven Jacobson

Rohit K. Jangra

Theodore S. Jardetzky

Dong-Yan Jin

Hailing Jin

Tao Jin

Stephen Anthony Kania

Kylene Kehn-Hall

Melissa M. Kendall

Linda J Kenney

Tammy L Kielian

David M. Knipe

Anna Konovalova

Jeroen Koomen

Christian Kost

Joel E. Kostka

Ákos T. Kovács

Richard J. Lamont

Helen M. Lazear

Mary-Cathrine Leewis

David A. Leib

Stanley M. Lemon

Daniel J. Lessner

Daisy W. Leung

Petra Anne Levin

Asaf Levy

Kim Lewis

Zachary A. Lewis

Dominique H. Limoli

Steven E. Lindow

Shan-Lu Liu

Marcelo Lopez-Lastra

Robert Luallen

Jeremy Luban

William Ludington

Robbie B. Mailliard

Elizabeth K. Mallott

Alexander Mankin

Ruben A. T. Mars

Kerrie L. May

Shonna M. McBride

John P. McCutcheon

Katherine McMahon

Joshua Chang Mell

Laura A. Mike

Makedonka Mitreva

Mary Ann Moran

Nancy A. Moran

Evangelia Morou-Bermudez

Poonam Mudgil

Indira U. Mysorekar

William Wiley Navarre

Stuart J. Neil

Michinaga Ogawa

Marco Rinaldo Oggioni

Molly Ohainle

George O'Toole

Michael Otto

John S. Parker

Colin R. Parrish

Andrew Pekosz

Alexandre Persat

Margaret Phillips

Mariana G. Pinho

Mihai Pop

Read Pukkila-Worley

Tracy Lyn Raivio

Glenn Rall

William Ratcliff

Kasie Raymann

Roland R. Regoes

Gemma Reguera

Markus W. Ribbe

Jeremy M. Rock

Antonis Rokas

Jeroen P. J. Saeij

Hassan Salem

Maria Sandkvist

Peter Sarnow

Karin Sauer

Jeffrey W. Schertzer

Bernhard H. Schink

Patrick D. Schloss

Ruth Anne Schmitz

John Schoggins

Robert T. Schooley

Leon N. Schulte

Drew Joel Schwartz

Jessica A. Scoffield

Pascale Serror

Amit Sharma

Bang Shen

Yuta Shirogane

Sujan Shresta

Steven P. Sinkins

Nicolas Sluis-Cremer

Kenneth Söderhäll

Peter Solomon

Joseph A. Sorg

Sujatha Srinivasan

Gary Stacey

Lisa Y. Stein

Mark D. Stenglein

Blaire Steven

Garret Suen

Motoyuki Sugai

Michael G. Surette

Curtis A. Suttle

Nick Talbot

Liang Tong

Eva M. Top

Ralph A. Tripp

Raphael Valdivia

Jan Roelof van der Meer

Andres Vazquez-Torres

Joe Vogel

Joseph Thomas Wade

William G. Wade

Honorine D. Ward

Christopher M. Waters

Brian Weiss

Matthew D. Weitzman

Ronald C. Wek

Paula Welander

Joel O. Wertheim

Rachel Whitaker

Elizabeth A. White

Kelly C. Wrighton

Yongzhen Xia

Jin-Rong Xu

Joanne Yew

Ozlem Yilmaz

Natalya Yutin

Joseph Zackular

Liping Zhao

